# *COMT* and *DRD2/ANKK-1* gene-gene interaction account for resetting of gamma neural oscillations to auditory stimulus-driven attention

**DOI:** 10.1371/journal.pone.0172362

**Published:** 2017-02-21

**Authors:** Manuel Garcia-Garcia, Marc Via, Katarzyna Zarnowiec, Iria SanMiguel, Carles Escera, Immaculada C. Clemente

**Affiliations:** 1 Institute of Neurosciences, University of Barcelona, Barcelona, Spain; 2 Brainlab-Cognitive Neuroscience Research Group, Department of Clinical Psychology and Psychobiology, University of Barcelona, Barcelona, Spain; 3 Institut de Recerca Sant Joan de Déu (IR-SJD), Barcelona, Spain; Duke University, UNITED STATES

## Abstract

Attention capture by potentially relevant environmental stimuli is critical for human survival, yet it varies considerably among individuals. A large series of studies has suggested that attention capture may depend on the cognitive balance between maintenance and manipulation of mental representations and the flexible switch between goal-directed representations and potentially relevant stimuli outside the focus of attention; a balance that seems modulated by a prefrontostriatal dopamine pathway. Here, we examined inter-individual differences in the cognitive control of attention through studying the effects of two single nucleotide polymorphisms regulating dopamine at the prefrontal cortex and the striatum (i.e., *COMTMet108/158Val* and *ANKK1/DRD2TaqIA*) on stimulus-driven attention capture. Healthy adult participants (N = 40) were assigned to different groups according to the combination of the polymorphisms *COMTMet108/158Val* and *ANKK1/DRD2TaqIA*, and were instructed to perform on a well-established distraction protocol. Performance in individuals with a balance between prefrontal dopamine display and striatal receptor density was slowed down by the occurrence of unexpected distracting events, while those with a rather unbalanced dopamine activity were able maintain task performance with no time delay, yet at the expense of a slightly lower accuracy. This advantage, associated to their distinct genetic profiles, was paralleled by an electrophysiological mechanism of phase-resetting of gamma neural oscillation to the novel, distracting events. Taken together, the current results suggest that the epistatic interaction between *COMTVal108/158Met* and *ANKK1/DRD2 TaqIa* genetic polymorphisms lies at the basis of stimulus-driven attention capture.

## Introduction

The accurate control of attention requires a precise balance between maintaining stable mental representations of relevant information in working memory and, at the same time granting access of potentially relevant unexpected stimuli to the focus of consciousness. Yet, the genetic underpinnings of this balance have not been established. A large body of evidence supports the role of the dopamine (DA) neurotransmitter system in the control of attention, both goal-[1–4], and stimulus-driven [5–7]. In particular, DA stimulation in prefrontal cortex (PFC) promotes stability of representations by increasing distracter resistance [8], whereas DA D2 receptor (DRD2) binding, particularly found at the striatum [9], facilitates the updating of new mental representations [10]. Accordingly, a current model proposes that the updating of working memory contents hinges on a subtle balance between the active maintenance of stable task representations and the flexible updating of those representations [7]. This model draws an inverted-U function between DA transmission and updating operations in working memory according to which working memory updating is optimal within a limited range of DA transmission [11–13]. More recently, Tian et al (2013) reported a U-shaped modulation by dopamine signaling in different components of the salience network using functional connectivity density mapping, suggesting a functional system-dependent modulation of dopamine signaling [14]. However, the role of this model of DA function on the stimulus-driven control of attention, such as that triggered by environmental novel events [15] has not yet been determined.

Here, we examined the epistatic interaction of two DA-related single nucleotide polymorphisms (SNPs) during stimulus-driven attention. For the regulation of PFC DA concentrations, the Val158Met SNP in the cathecol-O-methyltransferase gene (*COMT*) was selected due to its role in inactivating the DA diffused out of the synaptic cleft in the PFC [16]. The substitution of Met by Val is thought to increase the efficiency of the enzyme [17], so Val homozygous individuals are thus expected to have decreased stability of mental representations [16,18]. On the other hand, a SNP in the ankyrin repeat and kinase domain containing 1 gene (*ANKK1TaqIA*) was studied, as TaqIA1 carriers show a 30–40% reduction in DRD2 density, and therefore, lower DRD2 binding [19]. Recent reports have suggested *COMT* and *DRD2/ANKK1* gene interactions in other complex cognitive tasks such as working memory [20,21] and in the functional connectivity of different resting-state networks [14,22].

Here we tested the hypothesis that individuals with a putative balance between prefrontal dopamine availability and D2 receptor density (i.e., MetA1- ValA1+) would be prone to behavioral distraction as a result of stimulus-driven attention towards unexpected but potentially relevant novel events [23] relative to individuals included in the unbalanced groups (i.e., ValA1-, MetA1+). The neural mechanisms underpinning stimulus-driven attention were examined through brain responses in the gamma frequency band. We previously found that an increase in the phase-coherence of gamma band responses (GBRs) was associated with brain mechanisms of increased attention [24]. Similar results had been observed in the neurons activated by an attended stimulus in macaques using local field potentials [25]. Moreover, there is increasing evidences that dopamine profiles are implicated in the modulation of cortical gamma band synchrony and variation in the *COMT* gene has been associated to differences in gamma brain oscillations during working memory [26,27].

## Materials and methods

### Participants

Forty subjects (all Caucasian, six men, two left-handed, mean age 22 ± 4.2 years, range 18–29 years) participated in the present study. They were selected from a wider sample of volunteers in which the two genotypes of study were in Hardy-Weinberg equilibrium. All participants were interviewed through an adapted version of the Clinical Interview of the Diagnostic and Statistical Manual (DSM IV-R), for exclusion of subjects with neurological and psychiatric illness, phobias, and drug consumption. All subjects provided written informed consent at each phase of the study (interview, buccal cells extraction and EEG recordings). The study was conducted in compliance with the principles of the Declaration of Helsinki and was reviewed and approved by the Bioethics Committee of the University of Barcelona. All participants had normal audition and normal or corrected-to-normal vision.

We examined the epistatic interaction of two DA-related SNPs during task performance. For the regulation of PFC DA concentrations, the Val158Met SNP in the cathecol-O-methyltransferase gene (*COMT;* rs4680; [28] was examined, due to the role of such enzyme in inactivating the DA diffused out of the synaptic cleft in the PFC [16]. The substitution of Met by Val is thought to increase the efficiency of the enzyme [17], so Val homozygous individuals are thus expected to have decreased synaptic PFC DA levels relative to Met homozygous individuals and subsequent decreased stability of mental representations [16,18]. On the other hand, a well-studied SNP designated TaqIA consisting in a glutamate-to-lysine substitution within the gene named ankyrin repeat and kinase domain containing 1 (*ANKK1;* rs1800497) and lying 10 kb downstream the coding region of the DRD2 [29] was studied. TaqIA1 carriers show a 30–40% reduction in DRD2 density as compared to A2 homozygous as shown by in vitro [30] and *in vivo* studies [31,32]. A1 carriers display, therefore, lower DRD2 binding [19].

After exclusion by diagnostic criteria and obtaining the *COMT Val*158Met and *ANKK1/DRD2* TaqIA polymorphisms, the participants homozygous for the *COMT* (Met/Met, Val/Val), and those presenting the most frequent alleles for *ANKK1/DRD2* (A1, A2) were selected for an EEG recording session. Participants genotyped as Met/Met were assigned to the MetA1+ group when they carried the A1 allele (A1/A1, or A1/A2), and to the MetA1- group when they were homozygous for the A2 allele. Participants genotyped as Val/Val were assigned to the ValA1+ group when they carried the A1 allele (A1/A1, or A1/A2) and to the ValA1- group when they were homozygous for the A2 allele. We discarded EEG recordings from seven participants in which less than 70% of the trials could be retained for analysis after artifact rejection. From the remaining 33 individuals, seven composed the MetA1+ group, nine the MetA1- group, seven the ValA1+ group, and ten were included in the ValA1- group. Participants from each of the genetic groups did not differ significantly in age, state or trait anxiety scores (STAI, [33].

### DNA isolation and genotyping

Cheek cell swabs were used to collect biological samples and DNA was extracted using the Epicentre^®^ BuccalAmp^™^ DNA Extraction Kit (Epicentre, Madison, WS). TaqMan SNP Genotyping Assays (Applied Biosystems, Foster City, CA, USA) were used to determine individual alleles at the *COMT* Val158Met and *ANKK1/DRD2* TaqIA genetic variants.

### Stimuli and procedure

Participants performed a modified version of a well-characterized auditory-visual distraction task [23,34–36] with two conditions: a 1-back working memory (WM) condition and a 0-back condition with no WM load, as adapted for previous studies [37,38]. Each trial lasted 1300 ± 300 ms and was formed of a visual target preceded in 300 ms by an auditory stimulus. Participants were instructed to respond to visual stimuli as fast and accurately as possible and to ignore the auditory stimulation. The auditory stimuli were presented through Sennheiser^®^ HD202 headphones and consisted of a 600 Hz standard tone with an intensity of 90 dB (200 ms duration) in 80 percent of the trials and a unique environmental complex novel sound in the remaining 20 percent of the trials. Novel-sound trials were always preceded by at least one standard tone trial. Novel sounds were 100 unique sounds such as those produced by a drill, hammer, rain, door or telephone ringing, selected from a larger pool [34] as the more easily identifiable [36], and with similar spectrotemporal features [39]. Four blocks of 250 trials were delivered, there being two blocks of each condition. The order of these four blocks, as well as the order of the conditions was counterbalanced across subjects. Visual stimuli were single digits (1–4 and 6–9) presented on a screen for 200 ms in white color against a black background subtending a vertical angle of 1.53° and a horizontal angle of 2.10°. In the WM0 condition, participants had to decide by a button press whether the digit presented was larger or smaller than five. The WM1 condition consisted of a 1-back task in which participants had to decide whether the digit presented was larger or smaller in value than the digit presented in the previous trial. All participants responded with the middle and index fingers with the same hand for larger and smaller respectively. Before the experiment, participants performed a five minute practice block for each condition in which the sound was turned off; the practice sessions were repeated until a minimum accuracy of 75 percent was reached. A schematic of the task is included in the supporting files.

### EEG data acquisition

EEG data was acquired following previously described procedures [40]. Sixty-four scalp electrodes were used to record (ANT Software b.v., Enschede, The Netherlands) electroencephalographic activity during task performance following the extended 10/10 convention in an acoustically and electrically shielded room. We obtained electro-oculographic (EOG) recordings with electrodes placed at the outer cantus of the right eye (horizontal EOG) and above the right eye (vertical EOG). The ground electrode was located on the chest and the reference was placed on the tip of the nose. The EEG was amplified and a sampling rate of 512 Hz was used to digitize the signal. We kept impedances below 10 kΏ during the recording session, which lasted about 25 minutes.

### Data processing

Data processing was performed with ASA 4.5.1.0 software (ANT^®^, Enschede, The Netherlands). For single sweep analysis, time epochs were defined as the time window starting 250 ms before and lasting until 1000 ms after auditory stimulus onset (i.e., 700 ms from visual stimulus onset), with the pre-stimulus period used as a baseline. We applied an independent component analysis (ICA) for correcting eye movement and blink related artifacts as implemented in the ASA 4.5.1.0 software [41]. Subsequently, epochs still contaminated with ocular movements or muscle artifacts were rejected by an automatic artifact rejection procedure if their peak-to-peak amplitude exceeded 150 μV. For analysis of the GBR, the EEG signal was band-pass filtered between 30 and 60 Hz. To equalize the number of sweeps for better control of possible signal-to-noise ratio differences, all novel sweeps and a similar number of randomly selected standard sweeps were taken from each condition. For each participant an average of 74.9 trials per condition were used for analysis.

### Data analysis

To obtain the time-frequency components from the gamma range, the signal was decomposed by means of a continuous Wavelet transform. Time-frequency transforms were obtained by the application of complex-valued Morlet wavelets, which are Gaussian in both the time and frequency domain. Complex Morlet wavelets *w* can be generated in the time domain for different frequencies, *f*, according to the equation:

$$w(t,f)=A\text{exp}(-t^{2}/2\sigma_{t}^{2})\text{exp}(2i\pi ft),$$

where *t* is time, $A={\left(\sigma_{t}\sqrt{\pi }\right)}^{-1/2}$, *σ*_*t*_ is the wavelet duration, and $i=\sqrt{-1}$.

A ratio of f_0_/ σ_f_ = 12 was used, where f_0_ is the central frequency and σ_f_ is the width of the Gaussian shape in the frequency domain. The analyses were performed in the 30–60 Hz frequency range, with a central frequency at 0.75 Hz intervals (forty frequency steps). For different f_0,_ time and frequency resolutions can be calculated as 2σ_t_ and 2σ_f_, respectively. σ_t_ and σ_f_ are related by the equation σ_t_ *=* 1*/*(2πσ_f_).

In order to calculate the evoked activity (amplitude of GBRs), epochs were averaged for each condition and participant and then decomposed by means of a continuous Wavelet transform. Intertrial phase-coherence was calculated by means of the phase-locking of oscillatory activity measured using the phase-locking factor (PLF) [42]. This is a measure for phase identity across trials and is bounded between 0 (non-phase-locked signal) and 1 (phase-locked signal). The statistical significance of the distribution of phases was assessed by means of a circular statistics (Rayleigh test) with a threshold of p = 0.01. Since Rayleigh tests reached the significance threshold for every participant and channel, PLF values were used in further analyses. A 250 ms pre-stimulus time-widow (-250 to 0 ms) was used as baseline for the time-frequency information and the mean of this time window was subtracted for the time frequency matrix for each frequency and time point.

Maximal amplitude and PLF values of the GBRs were obtained in the latency windows from 100 to 200 ms, as GBRs are strongly synchronized in the first 100 ms after sensory stimulation and reflect very early stages of stimulus evaluation [43]. Because previous studies have referred 40 Hz activity to the increase of attention [24,44,45], analyses were performed for this frequency range around 40 Hz (35–45 Hz).

### Statistical analysis

The first five trials of each block, as well as those trials following a trial containing a novel sound were excluded from the analyses. A correct button press within 100–1200 ms after visual stimulus onset was regarded as a hit, and the mean response time (RT) was computed for hit trials only. Hit rate (HR) and RT were compared by means of three-factor repeated-measures ANOVA including the within-subject factors Novelty (standard, novel) and working memory load (with and without working memory load), and the between-subjects variables of the polymorphisms for the *COMT* (Met and Val) and the *ANKK1/DRD2* (A1+ and A1-) genes. Pair-wise *post hoc* comparisons were performed to significant interactions in order to elucidate which groups present a significant effect in any of the conditions.

For analysis of both amplitude and PLF of the GBR, maximal values in the defined latency window were measured for the auditory GBR for eighteen of the recorded channels (F3, Fz, F4, FC3, FCz, FC4, C3, Cz, C4, CP3, CPz, CP4, P3, Pz, P4, PO3, POz and PO4) at 35–45 Hz frequency range. Repeated measures of four-factor ANOVAs were performed including the factors Novelty (standard, novel), WM (with and without WM load), Region (six levels of frontality corresponding to F, FC, C, CP, P and PO) and Laterality (three levels for left, midline and right channels), and the between-subject variables of the polymorphisms for the COMT (Met and Val) and the DRD2 (A1+ and A1-). Greenhouse-Geisser correction of the degrees of freedom was applied, with the corrected *P*-values reported. Pair-wise *post hoc* comparisons were performed to significant interactions in order to elucidate which groups present a significant effect in any of the conditions.

All significant genetic effects were confirmed by means of permutation tests and Bonferroni multiple test correction to account for the potential occurrence of false positives due to the limited sample size. In the permutation tests, 2500 datasets were generated by randomly assigning genotypes to participants in the same proportions observed in the original dataset and performing the repeated-measures ANOVA (i.e. permuting genotypes and keeping all other variables). Then, we created the distribution of probabilities in the 2500 permutations and the two-sided probability of the permutation test was calculated as the proportion of permutations where the probability was smaller than or equal to the observed probability in the original dataset [46]. The Bonferroni multiple test correction was performed separately for each group of analyses (performance, amplitude of oscillatory activity, and phase-coherence of oscillatory activity) correcting the significance threshold by the number of tests performed.

All relevant data necessary to reproduce the statistics, tables, and graphs in the manuscript (performance and PLF datasets) are available in the supporting files.

## Results

### Performance

**Fig 1:**
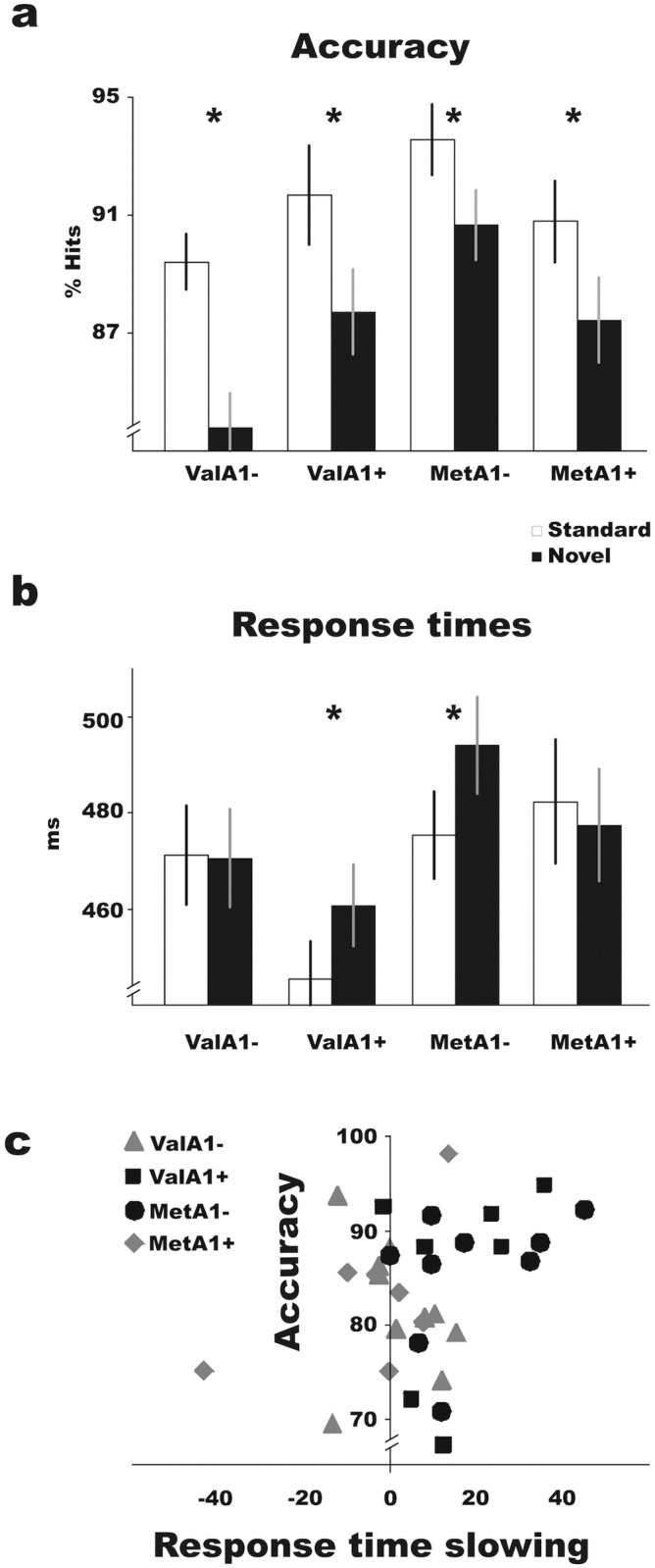
Task performance in all four groups. (**a**) Accuracy was lower in trials with novel sounds in comparison to those with standard sounds in all four groups (*, p<0.005). Notice the inverted-U shape of accuracy in the two trial types for the four groups arranged in the panel according to their levels of PFC-Striatum DA balance. (**b**) Response times in standard and novel trials (*, p<0.03). Notice that two groups displayed similar RT (i.e., ValA1- and MetA1+) in standard and novel trials, whereas the other two groups (i.e., ValA1+ and MetA1-) showed larger RT in novel than in standard trials. (**c**) Distribution of mean accuracy and RT slowing in all single individuals. Notice that the black symbols, representing individuals in the balanced groups, are mainly distributed on the upper right part of the plot, showing larger RT slowing after novel relative to standard sounds, but better mean accuracy than the unbalanced groups. In contrast, individuals from the unbalanced groups (white symbols) were distributed on the lower part of the graph as their accuracy was slightly worse, and along the Y axis because their RTs were not delayed by the novel sounds.

Mean performance accuracy (HR) was 83.9% with lower figures in the condition with working memory load relative to the one with no load (F_1,29_ = 104.88, p<0.001), as well as in novel than in standard trials (F_1,29_ = 60.62, p<0.001; Fig 1a). A larger accuracy decrease following novel sounds was observed in the WM1 than in the WM0 condition (F_1,29_ = 12.89, p = 0.001).

RTs were longer in the condition with working memory load relative to the one with no load (F_1,29_ = 11.47, p = 0.002) and were also delayed in novel relative to standard trials, indicating behavioral distraction (F_1,29_ = 9.85, p = 0.004; Fig 1b). Interestingly, a significant Novelty X *COMT* X *ANKK1/DRD2* interaction (F_1,29_ = 13.808, p<0.001, perm-p<0.001) revealed that ValA1- and MetA1+ groups had similar RT in standard and novel trials, whereas the ValA1+ and MetA1- groups displayed longer RTs following novel relative to standard sounds (ValA1+: F_1,6_ = 9.45, p = 0.022, perm-p = 0.015; MetA1-: F_1,8_ = 13.20, p = 0.007, perm-p = 0.003; Figs 1b and 3). No main effect was found on RT or accuracy for *COMT* or *ANKK1/DRD2* groups independently. In addition to remaining significant in the permutation tests, all associations with performance (genetic and non-genetic) remained significant after a Bonferroni correction for multiple testing, with the exception of the *post hoc* analysis of ValA1+ on RT (p>0.0125).

Balanced and unbalanced groups displayed different patterns of speed-accuracy tradeoffs when they were confronted with distracting novel sounds. As shown in Fig 1c, individuals in the balanced groups (MetA1- and ValA1+) showed larger RT delays after novel relative to standard sounds, but better mean accuracy than the unbalanced groups. In contrast, individuals from the unbalanced groups (MetA1+ and ValA1-) had slightly worse accuracy but their RTs were not delayed by the novel sounds. As no interaction was found between the effect of novel sounds on response time and the conditions with and without working memory load, we collapsed the data from the two working memory conditions for graphical purposes, and this factor will not be discussed further.

### Gamma band responses

#### Amplitude of oscillatory activity

Maximal values of oscillatory evoked activity around 40 Hz were analyzed for latencies between 100–200 ms after auditory stimulus onset. A main Novelty effect (F_1,33_ = 64.9, p<0.001) revealed larger amplitudes in novel relative to standard trials. All four groups showed this increase in evoked activity in novel compared to standard trials (ValA1-: F_1,12_ = 28.8, p<0.001; ValA1+: F_1,6_ = 15.3, p = 0.008; MetA1-: F_1,9_ = 12.7, p = 0.006; MetA1+: F_1,6_ = 29.0, p = 0.002). All associations with oscillatory activity remained significant after a permutation test and Bonferroni correction for multiple testing (p<0.01).

#### Phase-coherence of oscillatory activity

**Fig 2:**
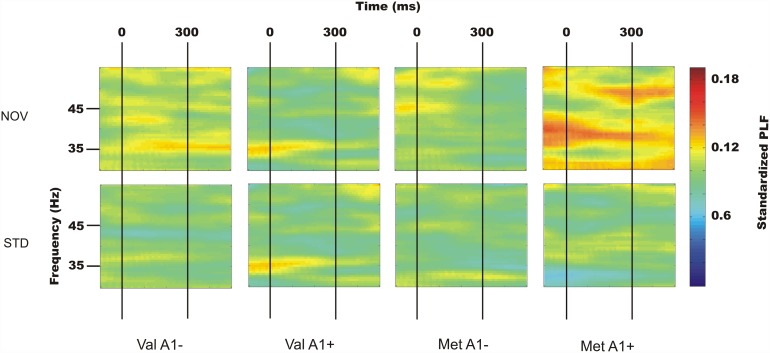
Standardized PLF values for novel and standard trials. Plots of standardized PLF values for novel and standard trials are shown for all four groups’ Cz for frequencies from 30 to 55 Hz. ValA1- and MetA1+ groups showed enhanced PLF in novel compared to standard trials around 100 ms post-sound onset, whereas ValA1+ and MetA1- groups had similar PLF values to both stimulus types.

**Fig 3:**
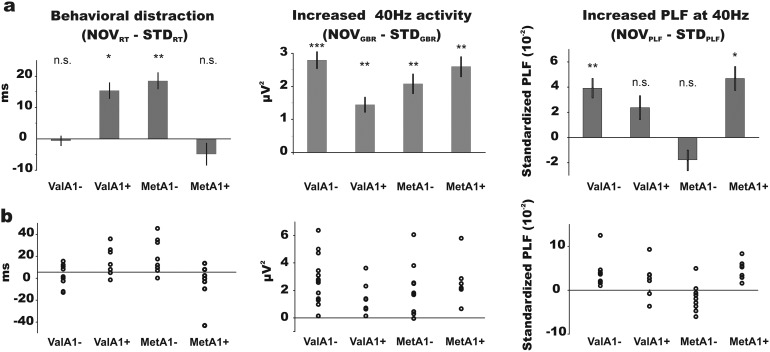
RT, amplitude and PLF of 40 Hz oscillatory activity for novel relative to standard trials. (**a**) Left panel shows the mean RT for novel compared to standard trials; notice that only the ValA1+ and MetA1- groups had larger RT for novel as compared to standard trials. Middle panel shows the increase in amplitude of neural oscillations at 40 Hz locked to novel sounds compared to that locked to standard sounds at Cz. Notice that amplitudes were larger after novel compared to standard sounds in all four groups. Right panel shows the increase of the PLF of neural oscillations at 40 Hz locked to novels sounds relative to that locked to standard sounds at Cz. Notice that PLF was larger after novel compared to standard trials in only the ValA1- and MetA1+ groups. (**b**) Scatter plots of individual values for RT (left panel), amplitude of the evoked 40 Hz oscillatory activity (middle panel), and PLF of the 40 Hz oscillatory activity (right panel). (*, p<0.05; **, p<0.01; ***, p<0.001; n.s., p>0.05).

Phase-coherence was analyzed through the phase-locking factor (PLF) of the 40 Hz brain activity between 100–200 ms from sound onset (Fig 2). A main Novelty effect (F_1,29_ = 7.52, p = 0.010) revealed larger PLF values in novel sound trials as compared to standard sound trials. Remarkably, a significant Novelty x *COMT* x *ANKK1/DRD2* interaction (F_1,29_ = 4.68, p = 0.039, perm-p = 0.039) revealed larger PLF values in novel relative to standard trials in ValA1- (F_1,9_ = 14.03, p = 0.005, perm-p = 0.002) and MetA1+ groups (F_1,6_ = 6.95, p = 0.039, perm-p = 0.028), but similar values for standard and novel trials in the ValA1+ and MetA1- groups (Figs 2 and 3). We did not observe any effect of electrode region or laterality on PLF values. The distribution of PLF values across the scalp is depicted in the supplementary materials. All genetic associations were confirmed after 2500 permutations. However, these differences were only significant among ValA1- carriers after Bonferroni correction (p<0.01) due to the limited sample size.

## Discussion

The current study explored the epistatic interaction of SNPs in the *COMT* and *ANKK1/DRD2* genes regulating the prefrontostriatal DA system on stimulus–driven attention. ValA1+ and MetA1- individuals, associated to a balance between PFC DA levels and DRD2 density, experienced a delay in RT after the occurrence of novel events in comparison to standard sounds, whereas individuals with presumably unbalanced prefrontostriatal DA patterns (ValA1- and MetA1+) showed similar RT in novel and standard trials. The amplitude of neural oscillations at 40 Hz evoked to the auditory stimuli increased in novel relative to standard trials in all groups similarly. However, while sound-locked oscillations showed similar inter-trial phase-coherence for both auditory stimuli in ValA1+ and MetA1- groups, the phase-coherence of 40 Hz neural oscillations increased in novel relative to standard trials for ValA1- and MetA1+ individuals.

*COMT* Val158Met and *ANKK1/DRD2* TaqIA interaction played a critical role in behavioral distraction. Involuntary attention capture is an ecologically critical mechanism for detecting potentially relevant stimuli in the environment and is expected to occur in all individuals even in the absence of RT delays. In fact, larger amplitudes of oscillatory activity at 40 Hz were observed in novel relative to standard trials in all groups, indicating increased attention allocated to novel events in all participants [44,47–52]. However, *COMT* and *ANKK1/DRD2* gene-gene interaction seemed to draw differences in the behavioral and electrophysiological concomitants of that increase of attention. While possession of Met and lack of A1 allele (i.e., MetA1-) and possession of Val and A1 alleles (i.e., ValA1+) are expected to display a balance between DA levels and DRD2 density, the other combinations of these polymorphisms would result in an unbalanced distribution of DA display in PFC and striatum. While the former experienced distraction through a RT delay following novel sounds, the unbalanced groups did experience the behavioral distraction effect, but through a general accuracy decrease. The impulsive behavior suggested by ValA1- performance fits into the model of Cools (2008) and Cools and D'Esposito (2011) [7,53]. According to this model, ValA1- would be expected to display lower PFC DA and show thus more cognitive flexibility, leading them to integrate the novel sound at the cost of a high accuracy decrease. On the other hand, MetA1+ individuals, expected to show a rigid behavior, showed higher resistance to interference and thus less of a distraction effect, as shown by lower accuracy decrease in novel trials accompanying the lack of RT delays, as compared with ValA1- individuals. Accordingly, the MetA1+ were the best performers of all four groups in a Stroop task [54], showing the best ability to inhibit interference.

These results map well on the models postulating an antagonistic effect between stability and flexibility in the PFC function [7,13,16,53]. High overall DA activity is expected for the balanced groups (MetA1-, ValA1+) [55]. This is beneficial for stable maintenance and manipulation of WM contents [20], in accordance with their slightly higher accuracy in the current task. However, high overall DA impairs the flexible switching between representations required in the Stroop task [54]. Accordingly, balanced groups lacked the flexibility needed to effectively avoid interference by unexpected novel events. Thus, they were slowed down by processing novel events as a counterpart to higher stability of maintenance and manipulation of goal-directed working memory contents. Because DRD2 binding leads to the update of mental representations [10], the combined effect of COMT activity and DRD2 density might reflect the interaction between a mechanism of maintenance of mental representations and a gating mechanism that allows the updating functions needed for activating new mental representations.

Nevertheless, while EEG amplitudes reflect rather sustained activation patterns of large cortical patches, the phase of brain oscillatory activity has been related to high temporal preciseness of particular neural firing patterns [56]. Therefore, the increased phase-coherence for novel relative to standard sounds experienced by the ValA1- and MetA1+ suggests a synchronizing mechanism of neural firing to novel events [57] during stimulus-driven attention ruled by DA neurotransmission. Phase resetting of neural oscillatory activity appear to set a mechanism to avoid behavioral distraction during the evaluation of potentially relevant events, while oscillatory activity related to the evaluation of these novel events might be smeared along those 20 ms of RT delay observed in the balanced groups

Moreover, the current results establish an inverted-U function of PFC DA activity and behavioral distraction by a novel event, by which middling levels of PFC DA activity lead to a delay in RT to the task while shifting attention towards novel events [13] (see Fig 3). An advantage in working memory maintenance and manipulation seem to result in a disadvantage for flexibly evaluating task-irrelevant novel events without slowing down the response to the current task. It is interesting to notice that while ValA1+ and MetA1- groups showed better performance in working memory tasks [20], ValA1- and MetA1+ were able to process novel events with no time cost (with however higher accuracy cost). This fact can clarify why some alleles that seem to provide disadvantage or even increase risk to develop psychiatric disorders survive evolution, as they provide advantages in complementary processes. This inverted-U function has recently been observed in the modulation of cortical gamma band synchrony by dopaminergic activity in computational and empirical investigations [26].

The present study did not assess the potential gender effects of the gene-gene interaction on contextual updating of mental representations. In spite of the small sample, the current results are robust to permutation tests and to multiple testing corrections and are suggestive of an epistatic interaction between *COMT* and *ANKK1/DRD2* genetic polymorphisms in the stimulus-driven control of attention. These results support previous claims of interaction between these genes in other complex cognitive tasks such as visual working memory [21] and in differences in connectivity of several resting-state networks [14,22]. The involuntary control of attention seems to be highly influenced by differential gene expression in fronto-striatal circuits, since a recent report has shown that genetic variation in the *BDNF* gene affects performance in an auditory distraction paradigm similar to ours [58]. Furthermore, the reported electrophysiological correlates of the control of attention might constitute a reliable endophenotypic marker of the DA system, and could help to isolate in near future dysfunction in the human DA system.

## Supporting information

S1 Fig Schematic of the task. (TIF)

S2 Fig Topographic plots of the distribution of PLF values across the scalp. PLF values in the eighteen channels analyzed (F3, Fz, F4, FC3, FCz, FC4, C3, Cz, C4, CP3, CPz, CP4, P3, Pz, P4, PO3, POz and PO4) were averaged across individuals in the same genotype group for the Novel and Standard stimulus conditions.
(PDF)

S1 Dataset Performance results. (XLSX)

S2 Dataset Phase-Locking Factor (PLF) results. (XLSX)
