# Supplementary figures and images for: COMT and DRD2/ANKK-1 gene-gene interaction account for resetting of gamma neural oscillations to auditory stimulus-driven attention
Source: PLoS One. 2017 Feb 21;12(2):e0172362. doi: 10.1371/journal.pone.0172362 (PMC5319755; doi:10.1371/journal.pone.0172362)

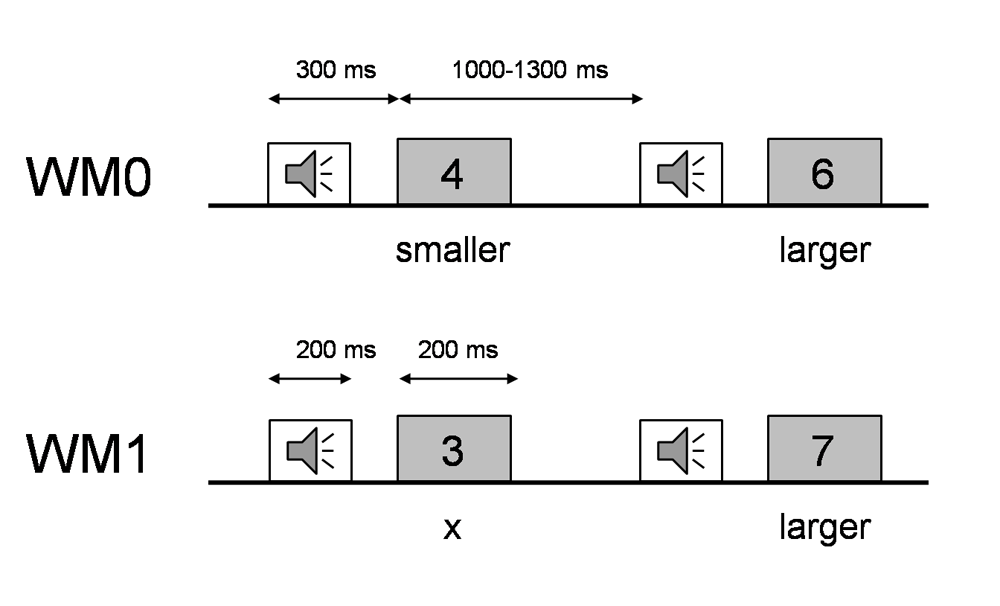

Supplement: S1 Fig — (TIF) [file pone.0172362.s001.tif]

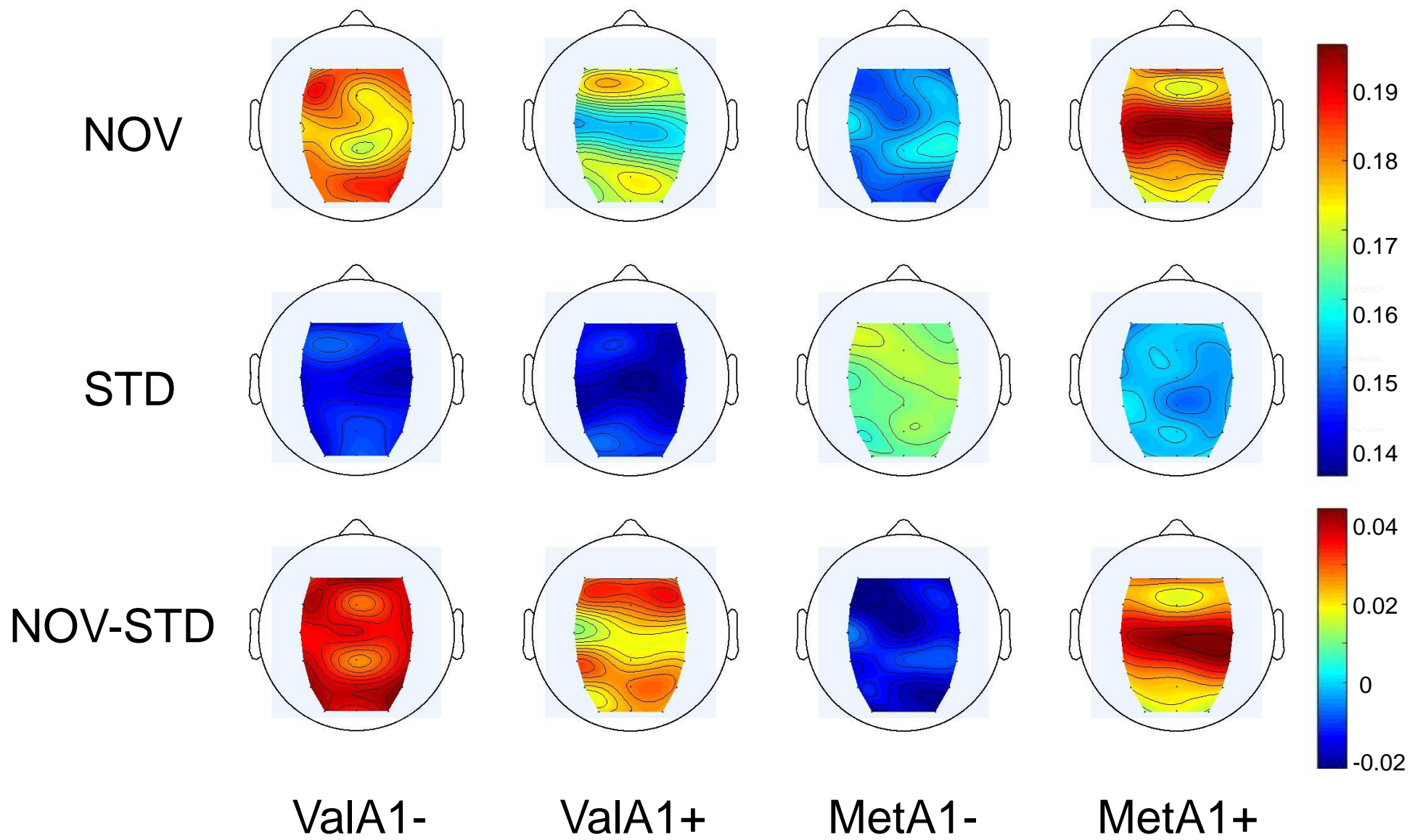

Supplement: S2 Fig — PLF values in the eighteen channels analyzed (F3, Fz, F4, FC3, FCz, FC4, C3, Cz, C4, CP3, CPz, CP4, P3, Pz, P4, PO3, POz and PO4) were averaged across individuals in the same genotype group for the Novel and Standard stimulus conditions. (PDF) [file pone.0172362.s002.pdf]
